# Serine Protease-Mediated Cutaneous Inflammation: Characterization of an Ex Vivo Skin Model for the Assessment of Dexamethasone-Loaded Core Multishell-Nanocarriers

**DOI:** 10.3390/pharmaceutics12090862

**Published:** 2020-09-10

**Authors:** Janna Frombach, Fiorenza Rancan, Katharina Kübrich, Fabian Schumacher, Michael Unbehauen, Ulrike Blume-Peytavi, Rainer Haag, Burkhard Kleuser, Robert Sabat, Kerstin Wolk, Annika Vogt

**Affiliations:** 1 Clinical Research Center for Hair and Skin Science, Department of Dermatology, Venereology and Allergy, Charité-Universitatsmedizin Berlin, Corporate Member of Freie Universitaet Berlin, Humboldt-Universitaet zu Berlin, and Berlin Institute of Health, 10117 Berlin, Germany; frombach@uni-potsdam.de (J.F.); fiorenza.rancan@charite.de (F.R.); katharinakuebrich@googlemail.com (K.K.); ulrike.blume-peytavi@charite.de (U.B.-P.); 2 Institute of Nutritional Science, University of Potsdam, 14558 Nuthetal, Germany; fabian.schumacher@uni-potsdam.de (F.S.); kleuser@uni-potsdam.de (B.K.); 3 Organic Chemistry, Institute of Chemistry and Biochemistry, Freie Universitaet Berlin, 14195 Berlin, Germany; m.unbehauen@fu-berlin.de (M.U.); haag@zedat.fu-berlin.de (R.H.); 4 Psoriasis Research and Treatment Center, Department of Dermatology, Venerology and Allergy/Institute for Medical Immunology, Charité-Universitaetsmedizin Berlin, Corporate Member of Freie Universitaet Berlin, Humboldt-Universitaet zu Berlin, and Berlin Institute of Health, 10117 Berlin, Germany; robert.sabat@charite.de (R.S.); kerstin.wolk@charite.de (K.W.)

**Keywords:** skin barrier, skin penetration, dermatotherapy, drug delivery, nanotechnology, core-multi shell nanostructure

## Abstract

Standard experimental set-ups for the assessment of skin penetration are typically performed on skin explants with an intact skin barrier or after a partial mechanical or chemical perturbation of the *stratum corneum*, but they do not take into account biochemical changes. Among the various pathological alterations in inflamed skin, aberrant serine protease (SP) activity directly affects the biochemical environment in the superficial compartments, which interact with topically applied formulations. It further impacts the skin barrier structure and is a key regulator of inflammatory mediators. Herein, we used short-term cultures of ex vivo human skin treated with trypsin and plasmin as inflammatory stimuli to assess the penetration and biological effects of the anti-inflammatory drug dexamethasone (DXM), encapsulated in core multishell-nanocarriers (CMS-NC), when compared to a standard cream formulation. Despite a high interindividual variability, the combined pretreatment of the skin resulted in an average 2.5-fold increase of the transepidermal water loss and swelling of the epidermis, as assessed by optical coherence tomography, as well as in a moderate increase of a broad spectrum of proinflammatory mediators of clinical relevance. The topical application of DXM-loaded CMS-NC or DXM standard cream revealed an increased penetration into SP-treated skin when compared to untreated control skin with an intact barrier. Both formulations, however, delivered sufficient amounts of DXM to effectively suppress the production of interleukin-6 (IL-6), interleukin-8 (IL-8) and Thymic Stromal Lymphopoietin (TSLP). In conclusion, we suggest that the herein presented ex vivo inflammatory skin model is functional and could improve the selection of promising drug delivery strategies for anti-inflammatory compounds at early stages of development.

## 1. Introduction

Preclinical disease models are essential tools for the evaluation of potential therapeutic targets, but also for the assessment of novel pharmaceutical formulations. The concept of reduction, refinement and replacement (3R) emphasizes the value of models derived from human skin cells and tissue to avoid unnecessary animal testing. Existing human skin models address different levels of biological complexity, ranging from human epidermis or full-skin equivalents reconstructed from keratinocytes to skin and organotypic culture set-ups [1,2,3,4]. Disease-specific features can be introduced in these systems by using patient-derived cells [5], by the modulation of disease-associated genes [6], by the addition of pathogens [7] or malignant cells [8], or by stimulation with disease-associated soluble mediators such as cytokines [9]. The required experimental set-up, however, is usually time-consuming, and the value of these models for penetration studies that specifically address penetration in diseased skin is limited, as they are typically hyperpermeable [10] and lack appendages and immune cells, all of which affect the tissue environment [11]. As a result, drug penetration assessments are typically performed on split-skin or full-thickness skin explants in diffusion cell chambers. However, ex vivo and in vivo investigations indicate that penetration across inflamed skin is more complex than expected and that standard barrier disruption models via the partial removal of the *stratum corneum* or chemical irritation do not sufficiently reflect those aspects [12]. As newer generations of drug delivery systems can be equipped with elements that address specific features of diseased skin [13,14], a closer investigation of the interactions of drug formulations with skin barrier components could provide important guidance for the selection of the most promising candidates.

Particularly in the field of nanotechnology, a wide range of different types of drug delivery systems has been developed to improve drug penetration and to enable controlled drug release, e.g., trilayer core-shell nanofibers for an accurate dual-stage drug release [15].

Furthermore, the establishment of skin models that more closely reproduce the features of diseased skin would also give the possibility to test drug biological activity and correlate it to their skin penetration. Among the various immunological and biochemical characteristics, enhanced serine protease (SP) activity is a common feature of inflamed skin [16,17,18,19,20]. The presence of an aberrant activity of SPs and their inhibitors in the *stratum corneum* and throughout the epidermis directly affects the biochemical microenvironment in superficial skin compartments, which are the first to come in contact with topically applied formulations [16,21,22]. SP activity exerts direct effects of keratinocytes, resulting in an altered proliferation, differentiation and desquamation [20] as a result of impaired lamellar body secretion, lipid processing and maintenance of corneodesmosomes [23,24], but it is also a key regulator of inflammatory cascades. One typical signaling pathway involves proteinase-activated receptors (PARs), membrane-spanning G protein-coupled receptors that get activated by the cleavage of an extracellular domain [25]. Among these, PAR1 and PAR2 have been discovered in various constitutive or transient cells in the epidermis, as well as in the dermis [26], where downstream signaling leads to the expression of various inflammatory mediators [27], such as interleukin-6 (IL-6) [28,29,30,31], interleukin-8 (IL-8) [30,32,33,34,35] and thymic stromal lymphopoietin (TSLP) [36,37,38,39].

Herein, we established an ex vivo short-term culture model of full-thickness human skin treated with low levels of trypsin and plasmin, following protocols adapted from mouse studies [40,41]. The skin physiological parameters and induction of inflammatory mediators were investigated. The model was created, because the use of SP as a biochemical stimulus could help analyze the penetration and efficacy of anti-inflammatory therapies. In order to obtain first insights as to whether cytokines stimulated by SP treatment could be regulated in such short-term cultures, we applied dexamethasone (DXM) encapsulated in core-multi shell nanocarriers (CMS-NC) or standard cream, two formulations which had been investigated in detail on intact ex vivo human skin [42,43] and reconstructed epidermis [44]. Among the broad range of available drug delivery systems, CMS-NC were chosen because of their established effectiveness for the delivery of a range of drugs across the skin barrier. CMS-NC are unimolecular structures composed of a branched core-unit [45] and amphiphilic polymeric chains that form a nonpolar inner shell and a hydrophilic outer shell [46]. When compared to solid lipid nanoparticles, CMS-NC were found to be more efficient in delivering low molecular weight hydrophilic compounds like Rhodamin B [47]. An improved delivery capacity, as compared to a cream formulation, was also found for the lipophilic dye Nile red [44]. Variants of CMS-NC have also been synthetized in order to make them biodegradable and less toxic [43,48,49]. Finally, the efficient delivery of DXM by CMS-NC was measured in ex vivo human skin with an intact barrier and after the removal of the *stratum corneum* layers by means of label-free X-ray spectromicroscopy [50], as well as in vivo in animal models of healthy, atopic and psoriatic skin [51,52].

## 2. Materials and Methods

### 2.1. Carrier Synthesis and Characterization

DXM was purchased by Merk Millipore.CMS-NC were synthesized as described earlier [44]. In short, hyperbranched polyglycerol amine (70% -NH_2_) was reacted with the N-hydroxysuccinimide ester (NHS-ester) of C18-mPEG_350_ in methanol. After dialysis, the nanocarrier (NC, which can be best described with the empirical formula hPG_10000_ [-NH_2_-C_18_-mPEG_350_]_0.7_) (CMS-NC) resulted. The detailed physicochemical characterization of this type of nanostructure has been described previously [53].

The NC used in this study consisted of a hyperbranched polyglycerol core that served as a focal point to which the amphiphilic double shell was attached. The double shell was formed by 90 amphiphilic linear residue aliphatic diacids, which made up the hydrophobic part, bound to a PEG chain terminated by a methoxy group as the hydrophilic part. To load DXM into the NC, the film uptake method was used, as described before [49]. In this method, a film of the DXM was prepared by evaporating a DXM-solution in a vial with a rotavap. Then, a stock solution of NC (10 g/L) was added, and after 22 h the resulting mixture was filtered through a 450 nm regenerated cellulose filter to remove any nonsolubilized DXM. Finally, the concentration of solubilized DXM was determined with high-performance liquid chromatography (HPLC), analogously to a previous publication [49]. A Gemini column (C18, 5 µm, 110 Å, 250 mm × 6.4 mm; Phenomenex, Torrance, CA, USA) was used. The eluent was 40% acetonitrile/60% H_2_O (*v*/*v*), with a flow rate of 1 mL/min, and a UV detector was employed (λ = 210 nm).

### 2.2. Serine Protease Treatment and Drug Application on Ex Vivo Human Skin

Intact excised abdominal human skin of healthy Caucasian volunteers was obtained from surgical plastic surgery and handled according to the Declaration of Helsinki Principles. Informed consent forms approved by the Institutional Ethics Committee of the Medical Faculty of the Charité–Universitaetsmedizin Berlin, Germany were signed (approval EA1/135/06, renewed on January 2018) by the donors. Defined treatment areas of 1 cm^2^ surrounded by 0.5 cm borders of untreated skin were used as non-SP controls or for SP treatment. To facilitate SP penetration, full-thickness excised skin still containing a thin layer of subcutaneous fat was fixed on a styrofoam block covered with aluminum foil and Parafilm. The samples were preincubated for 1 min with chloroform/methanol (2:1) (Merck KGaA, Darmstadt, Germany) on filter paper discs (12 mm^2^ diameter Finn Chambers^®^, SmartPractice, Hillerød, Denmark). This was then followed by a 3 µg/cm^2^ trypsin application (Merck KGaA) and intradermal injection of 46 µg/cm^2^ plasmin (Athens Research & Technology, Athens, GA, USA). The trypsin concentration of 3 µg/cm^2^ that was applied here, applied as 20 µL of a 150 µg/mL solution on the skin surface, was below the concentration used for cell culture passage or basal membrane digestion, e.g., 500 µg/mL (0.05%) for HaCaT keratinocytes. It was adapted from experimental protocols to make use of its proinflammatory properties. In cell cultures studies with keratinocytes exposed to 2.3 µg/mL trypsin, we identified this concentration as a moderate stimulus for the induction of the free radicals IL-6 and IL-8 [54,55].

Afterwards, the tissue samples were incubated in a humidified chamber at 37 °C, 5% CO_2_, 100% humidity for 16 h, followed by application of either phosphate buffered saline (Merck Millipore, Billerica, MA, USA) (negative control) or 10 µg/cm^2^ DXM formulated as standard cream (LAW-cream, 0.05% DXM, methyl-4-hydroxybenzoate, propyl-4-hydroxybenzoate, emulsified cetyl stearyl alcohol (Type A), glycerol 85%, isopropyl myristate, propylene glycol, edetate disodium, sorbic acid and purified water) or DXM encapsulated in CMS-NC (DXM loading 8.8% (*w*/*w*)) on a 1 cm^2^ skin surface and incubated for 24 h. In order to compare the developed model to a standard irritation model, some of the samples were treated with sodium lauryl sulfate (SLS, Roth, Karlsruhe, Germany), i.e., 20 μL/cm^2^ of 5% (*w*/*v*) SLS in deionized distilled water were applied topically using filter paper discs and removed after 4 h incubation. Incubation was continued for 12 h before DXM standard cream was applied, as described above. Skin samples were collected at t = 0 h, t = 16 h and t = 40 h (at the end of the total exposure period). From each tissue sample, four 0.5 × 0.5 cm blocks were cut and frozen until further processing for RNA, protein extraction or immunohistochemistry and immunofluorescence, respectively.

### 2.3. Protein Extraction

Protein was extracted from superficial *stratum corneum* (sSC) removed by cyanoacrylate skin surface stripping (CSSS), as described elsewhere [55]. Protein extracts from sSC were prepared using 1 × PBS with 10% ethanol (Merck Millipore) and 0.005% Tween 20 (Sigma Aldrich, Steinheim, Germany). CSSS-strips were incubated on ice for 3 h, followed by 10 min sonification in ice-cold water (Bandelin Sonorex RK102H, Bandelin electronic GmbH & Co. KG, Berlin, Germany), vortexed at 1800 rpm for 30 s and centrifuged at 450× *g* for 1 min. The total protein content was determined by a Pierce^™^ Coomassie Plus (Bradford) Assay Kit (ThermoFisher Scientific), and supernatants were stored at −80 °C.

The underlying skin was sectioned horizontally in 50-μm sections using a cryostat (Microm^™^ HM 560 ThermoFisher Scientific, Rockford, IL, USA). Sections of epidermis (100 μm from SC) and dermis (remaining 900 μm) were separated, based on a preassessment by optical coherence tomography (OCT) [56]. Protein extracts of epidermis and dermis were prepared using an extraction buffer containing 100 mM Tris (Trizma^®^ hydrochloride, Sigma Aldrich), 150 mM sodium chloride (Merck Millipore), 1 mM ethylenediaminetetraacetic acid (EDTA, Sigma Aldrich), 10% ethanol (Merck Millipore) and 1% Triton^™^ X-100 (Sigma Aldrich) in deionized water. Epidermis and dermis sections were homogenized in extraction buffer with a tissue lyser II (Qiagen GmbH, Hilden, Germany) for 1 min at 30 Hz, followed by incubation on ice for 45 min, sonification in an ice-cold water bath for 10 min and centrifugation at 450× *g* for 5 min. The total protein content was determined by a Pierce^™^ 660 nm Protein Assay Kit (ThermoFisher Scientific, Waltham, MA, USA), and the supernatants were stored at −80 °C.

### 2.4. Analysis of Protein Extracts

Extracts were investigated using human IL-6- and IL-8-CytoSet^™^ (CHC1263, CHC1303, Invitrogen Corporation, Waltham, MA, USA), human TSLP-ELISA Ready-SET-Go!^®^ Set (eBioscience, an Affymetrix company, Vienna, Austria), human IL1α-DuoSet^®^ELISA (R&D Systems, Minneapolis, MN, USA) and Profiler^™^ Arrays (Human Cytokine Array Panel A, ARY005, Human Chemokine Array, ARY017, Human Protease Array Kit, ARY021, R&D Systems, Minneapolis, MN, USA). All assays were performed according to the manual instructions provided by the manufacturer. DXM concentrations in the extracts were quantified by HPLC-MS/MS [42].

### 2.5. Gene Expression Analysis

Samples for RNA extraction were stored in RNAlater at −80 °C. The total RNA was extracted by an RNAeasy Fibrous Tissue Kit (Qiagen). The quantity and quality of RNA were checked using a NanoDrop 1000 Spectrophotometer (ThermoFisher Scientific) and by electrophoretic separation on denaturing agarose gel. For the TaqMan^TM^-based RT-qPCR analysis of IL-6, IL-8 and TSLP, a reverse transcription of RNA was performed using the QuantiTec Reverse Transcription Kit (Qiagen) in a thermal cycler. A qPCR analysis was carried out in a StepOnePlus™ device (Applied Biosystems, Waltham, MA, USA) using the Maxima Probe/ROX qPCR Master Mix (ThermoFisher Scientific) and ready-to-use detection assays for IL-6, IL-8, TSLP and hypoxanthine-guanine phosphoribosyltransferase (HPRT) as the house-keeping gene, containing double-labeled probes (Applied Biosystems, ThermoFisher Scientific). Based on identical amplification efficiencies and detected threshold cycle values, the gene expression was calculated relative to the HPRT expression, as described. [57] For RT-qPCR in an array setting, a reverse transcription of RNA was performed using the RT^2^ First Strand Kit (Qiagen) in a thermal cycler (GeneAmp PCR System 9700, Applied Biosystems^™^) prior to analysis by the human Inflammatory Response & Autoimmunity RT^2^ Profiler PCR Array (Qiagen) in a StepOnePlus™ device (Applied Biosystems). All assays were performed according to the supplier’s specifications.

### 2.6. Histology and Immunohistochemistry

Hematoxylin and eosin (HE) staining was performed following the manufacturer’s manual (Roth) on 5-µm longitudinal cryosections fixed with acetone for 10 min, and images were taken by optical microscope IX 50 (Olympus, Hamburg, Germany). For the immunohistochemistry, 5-µm cryosections were fixed with 4% paraformaldehyde (Sigma Aldrich) in PBS for 10 min and incubated with a DAKO^®^ protein block (Dako, Carpinteria, CA, USA) for 1 h at room temperature, followed by an incubation with three different primary monoclonal antibodies: mouse anti-human CD1A (clone: 10, Dako; at 1:100 in PBS for 1 h), mouse anti-human PAR2 (clone: SAM 11, Santa Cruz Biotechnology, Dallas, TX, USA; at 1:50 in PBS/5% bovine serum albumin for 1 h) or mouse anti-human Ki67 (clone: 4A1, Abgent, San Diego, CA, USA; at 1:100 in PBS/5% fetal calf serum (Biochrom, Berlin, Germany) for 2 h). A fluorescein-labeled horse antimouse IgG (H + L) antibody (Vector Laboratories, Burlingame, CA, USA; at 1:50 in PBS/5% fetal calf serum) was applied as a secondary antibody and incubated for 45 min. Sections were mounted in Vectashield^®^ Mounting Media with or without 4′,6-diamidino-2-phenylindole (DAPI, Vector Laboratories), and fluorescence images were taken by a BX 60 microscope (Olympus). Ki67-positive cells were counted in six randomly selected areas per slide using 10 slides per donor per time point.

### 2.7. Non-Invasive Skin Assessments

The following skin biophysical parameters of untreated and SP-treated skin were measured noninvasively during the 40-h incubation period in a humidified chamber at 37 °C, 5% CO_2_, 100% humidity: At t = 0 h, t = 16 h and t = 40 h, the transepidermal water loss (TEWL) was detected by a Tewameter^®^ TM 300, the pH value of the skin surface by Skin-pH-meter^®^PH 905 and the skin hydration by Corneometer^®^ CM 825 (Courage + Khazaka electronic GmbH, Germany). Optical coherence tomography (VivoSight, Michelson Diagnostics, Maidstone, Great Britain) was used for in situ imaging at 16 h and 40 h after treatment. The epidermis thickness was determined using VivoSight software tools (distance between the skin surface and basal membrane).

### 2.8. Statistical Analysis

Data obtained from >6 different donors were analyzed by a two-way analysis of variance (ANOVA), followed by a two-tailed student *t*-test (* *p* ≤ 0.05 and ** *p* ≤ 0.01). The data obtained from the experiments performed on the skin samples from <6 different donors, due to the limited availability of donor skin, are presented with descriptive statistics (mean, standard deviation).

## 3. Results

In order to assess the effects of the combined pretreatment on the skin explants, the skin barrier parameters typically measured in clinical trials were combined with histological evaluations as well as analyses of the mRNA expression and protein levels for inflammatory mediators in response to SP treatment and the topical application of two different DXM formulations.

**Figure 1:**
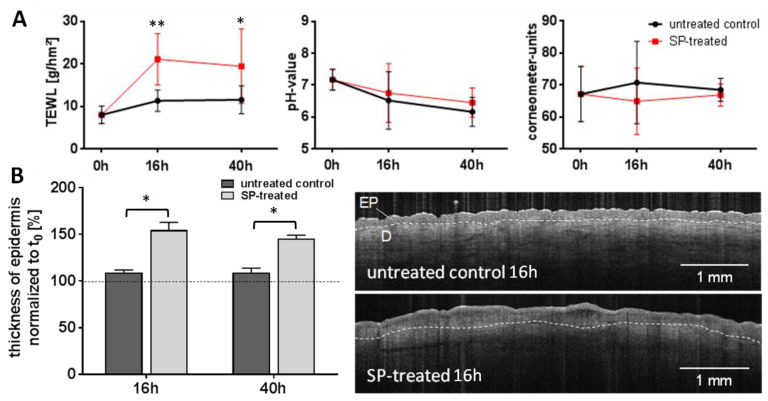
**Effects of SP treatment on skinbiophysical parameters.** Noninvasive measurement of the skin parameters of SP-treated ex vivo human skin compared to the untreated control during an incubation period of 40 h at 37 °C, 100% humidity and 5% CO_2_. (**A**) Transepidermal water loss (TEWL) increased in SP-treated skin, whereas the pH on the skin surface and skin hydration were not affected. (**B**) The thickness of the epidermis, measured in optical coherence tomography (OCT) images within the dotted line, increased after 16 h and 40 h of SP treatment. EP = epidermis, D = dermis. Data of all graphs are mean ± SD, n = six donors. Significances were determined by a two-way analysis of variance (ANOVA), followed by a two-tailed student *t*-test performed, where * *p* ≤ 0.05 and ** *p* ≤ 0.01 indicate statistically significant differences between the SP-treated skin and untreated control.

The effects of the serine protease-treatment on the skin barrier parameters, morphology and proliferation rate of ex vivo skin were as follows. In SP-treated skin, the rates of transepidermal water loss (TEWL) increased to about 2.5-fold of the initial value. The TEWL values of untreated ex vivo skin ranged between 8 and 12 g/m^2^h during the incubation period (Figure 1A), which largely corresponds to in vivo data, which typically vary between 5 g/m^2^h and 12 g/m^2^h in healthy subjects depending on sex, age and skin location [58,59]. No differences were observed with regard to pH and stratum corneum hydration. Specifically, the skin surface pH appeared to be of limited use in this experimental set-up. Baselines values were higher than under physiological in vivo conditions, most likely as a result of the skin treatment and exposure to tissue fluids during the surgery procedure. A thickening of the epidermis up to >150% of the initial value (mean [±SD] = 0.109 [±0.003] mm) in SP-treated skin was found by OCT measurements (Figure 1B).

**Figure 2:**
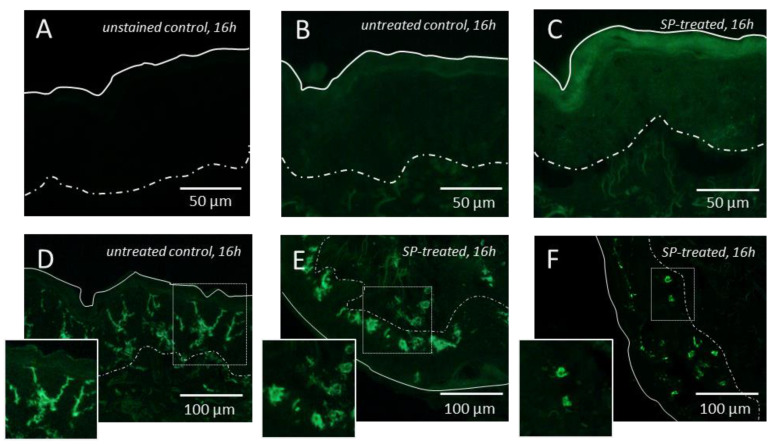
**PAR2 expression and Langerhans cell activation in SP-treated skin.** Representative images of sections stained for Protease activated receptor 2 (PAR2) compared to (**A**) the unstained control (**B**) show the presence of a PAR 2 signal in the upper part of the epidermis (**C**) and indicate an enhanced presence in SP-treated skin 16 h after pretreatment. (**D**–**F**) Representative images of sections stained for Cluster of Differentiation 1a (CD1a) show a loss of dendricity of CD1a+ epidermal Langerhans cells in SP-treated skin (representative images from n = 3 donors).

The histological assessment revealed no substantial morphological alterations of the untreated or SP-treated skin during the incubation period of 40 h (Supplemental Material, Figure S1). The cell proliferation rate, as quantified by the fraction of Ki67-positive cells, was not influenced by the SP treatment (Supplemental Material, Figure S2). However, SP-treated samples stained positive for PAR2, in accordance with the postulated mechanism of SP action (Figure 2A–C). The reduced number and dendricity of CD1a-positive epidermal cells in SP-treated skin indicated Langerhans cell activation and migratory activity (Figure 2D–F).

### 3.1. Expression of Inflammatory Mediators in Response to Serine Protease Treatment

**Figure 3:**
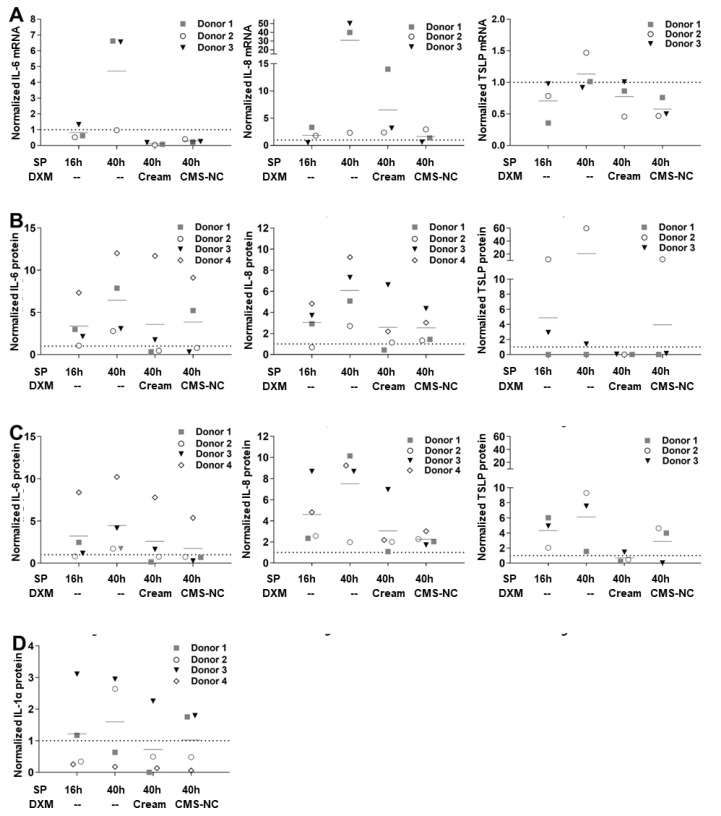
The relative expression of the proinflammatory markers IL-6, IL-8, TSLP and IL-1α in ex vivo human skin after the SP treatment shows the anti-inflammatory effects of DXM formulated in cream or encapsulated in CMS-NC. The mRNA and protein values were normalized to the untreated controls (non-normalized values are shown in the Supplemental Material, Figure S3). Short lines indicate the means of the different donors for each treatment group. Spotted lines indicate the expression level of the untreated controls. (**A**) mRNA expression of the investigated markers. The fold mRNA expression was first calculated with respect to the housekeeping gene hypoxanthine-guanine phosphoribosyltransferase (HPRT) and then normalized to the untreated controls. (**B**–**D**) Quantification of the investigated proteins in different skin layers (B = dermis, C = epidermis, D = superficial *stratum corneum*) measured by ELISA. The amounts of proteins were first calculated with respect to the total protein content of each extract and then normalized to the untreated controls.

To assess the functionality of the inflammatory ex vivo skin model in response to the SP treatment and topical application of anti-inflammatory formulations, the mRNA and protein levels of the proinflammatory mediators IL-6, IL-8, TSLP and IL-1α were investigated after 16 h and 40 h of SP stimulation and 24 h DXM treatment (Figure 3, Supplemental Material Figure S3). The expression levels of the chosen inflammatory markers were subjected to interindividual variability and corresponded to the positive controls treated with the irritant SLS (Supplemental Materials, Figure S3E–G).

The mRNA expression of IL-6, IL-8 and TSLP in the tissue (Figure 3A) was markedly enhanced after 40 h. The topical application of DXM formulations effectively downregulated the inflammatory response. This finding was supported by similar effects on the protein, both in the dermis (Figure 3B) as well as in the epidermis (Figure 3C). Changes in IL-1α in the sSC (Figure 3D) were less pronounced.

A wider profiling of cytokine and chemokine RNA and protein panels confirmed a mild-moderate increase of a range of mediators after SP treatment (Supplemental Material, Figure S4). The levels of individual cytokines and chemokines varied among the different donors. The pronounced inflammatory reactions of individual donors required a normalization to the baseline for each individual. Even then, no specific direction of the response towards atopic dermatitis- or psoriasis-like inflammation could be found, which is in accordance with the fact that the SP treatment is a broad stimulus (Supplemental Material, Figure S4).

### 3.2. Impact of Serine Protease-Treatment on Dexamethasone Penetration

**Figure 4:**
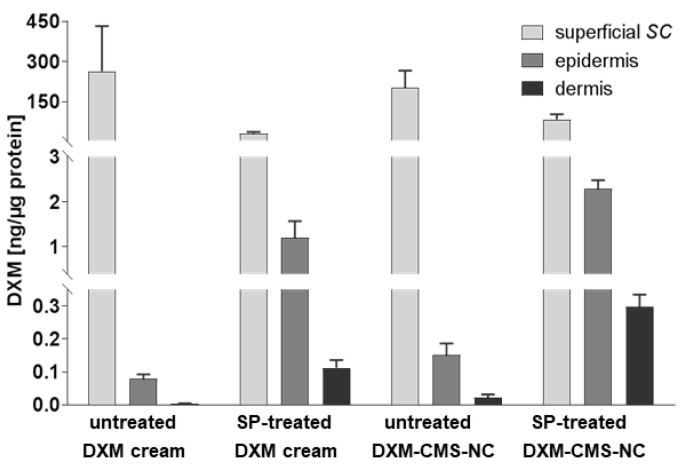
The SP treatment and CMS-NSC formulations increased the skin penetration of dexamethasone (DXM). The penetration of DXM after the topical application on the untreated and serine protease (SP)-treated ex vivo human skin was quantified in extracts of three different skin layers, superficial *stratum corneum* (s*SC*), epidermis and dermis, by HPLC-MS/MS. DXM concentrations were normalized to the total protein content of each extract. Data are shown as mean (+SD), n = 5 donors for the untreated samples and n = 3 donors for the SP-treated samples.

The impact of the SP treatment on drug penetration was assessed by a topical application of 10 µg/cm^2^ DXM on the skin sample surface, formulated in standard cream or in CMS-NC, on skin that was preincubated for 16 h with or without SP treatment. The drug concentrations in the tissue extracts, as determined by HPLC-MS/MS, revealed a marked increase of drug in the epidermis and in the dermis of SP-treated compared to untreated skin (Figure 4). Regardless of the skin condition, CMS-NC delivered higher amounts of DXM to deeper skin layers than standard cream did.

## 4. Discussion

In this study, we used SP administration to human skin explants as an inflammatory stimulus. The protocol was adapted from previous animal studies that showed that the topical application of trypsin on mice induced the development of atopic dermatitis-like features, including hyperproliferation and inflammation [40]. In another study, tissue inflammation that corresponded to psoriasis was induced by the intradermal injection of plasmin into murine skin [41]. In that sense, enhanced SP activity is a common characteristic of various inflammatory skin diseases. With regard to the assessment of novel drug delivery systems, it is a relevant factor because it affects barrier integrity, immune function and may also directly interfere with the carrier itself.

The proinflammatory effects of the herein applied stimulus proved to be mild to moderate, triggering inflammatory responses in the epidermis and dermis, as well as immune cell activation, without causing destructive changes in the epidermis morphology or a substantial decrease of the proliferative capacity. Given that the mechanical injury caused by the intradermal injection procedure is itself likely to contribute to the cytokine increase, we cannot conclude that the observed effects exclusively resulted from the epidermal and dermal SP addition. In previous investigations, however, Li et al. elegantly demonstrated the role of plasmin as an amplifier of skin inflammation using intradermal injections of phosphate-buffered saline compared to plasmin in murine skin [41]. The overall observation of the combined SP treatment being a moderate inflammatory stimulus is in accordance with our own experiments using human keratinocyte cultures, which showed that the addition of low concentrations of trypsin and plasmin resulted in a mild-to-moderate radical formation, as assessed by electron paramagnetic resonance (EPR), while also increasing IL-6 and IL-8 levels without impairing the overall cell viability [54]. In vivo, increased TEWL values, as observed in the culture model, were positively correlated with an enhanced activity of plasmin and trypsin-like KLKs in the *stratum corneum*. After topical treatment of murine skin with trypsin, TEWL increased by 35%. Trypsin-like proteases (including KLK5 and KLK14), tryptase-like plasmin, and urokinase activities were positively correlated with TEWL and negatively correlated with hydration, but chymotrypsin-like activities (including KLK7) were not. Interestingly, the type of protease activity that correlates with barrier integrity is consistent with the types of protease capable of activating PAR2, involved in barrier homeostasis [59], and the observed effects are in line with the values observed in diseased skin such as for AD patients [16,60,61], in whom up to three-fold TEWL increases are observed in severely affected lesional skin [62]. While swelling of the epidermis could just be the result of barrier disruption and maintenance in a humidified environment, the mRNA and protein analyses, along with the immunohistochemical staining, point towards SP-mediated inflammatory effects, e.g., PAR2 activation, as found in our model, typically initiates different inflammatory signaling pathways and induces the expression and secretion of various inflammatory mediators such as Intercellular Adhesion Molecule 1 (ICAM-1), IL-6, IL-8 and Granulocyte Macrophage-Colony Stimulating Factor (GM-CFS). In line with this, IL-6, IL-8 and (with a larger variability) TSLP were identified as markers that were suitable for assessing the response and subsequent anti-inflammatory activity of topically applied DXM in our model.

Despite their distinct clinical manifestations and immunological profiles, IL-6, IL-8 and Tumor Necrosis Factor-α (TNF-α) are elevated in both atopic dermatitis and psoriasis, as confirmed in tape strips from pediatric atopic dermatitis patients [63], TNF-α levels in stratum corneum extracts from psoriasis patients and cytokine analyses in epidermis and dermis [64]. Besides keratinocytes, fibroblasts and dermal mast cells are important sources, as shown for TNF-α in psoriasis and atopic dermatitis [65], and for IL-6 in psoriasis [66].

Similarly, Interleukin-8 is a potent chemotactic and proinflammatory cytokine, produced in the skin by a variety of cells in response to inflammatory stimuli. Recent studies suggest that, in addition to its potent actions on leukocytes, IL-8 exerts a direct influence on several functions of human epidermal cells. In the skin, a broad spectrum of cells, e.g., neutrophils, T-lymphocytes, mast cells, dermal macrophages, endothelial cells and keratinocytes, possess binding sites for IL-8 [67]. In our model, we addressed this aspect by combining topical and dermal protease addition. Thymic Stromal Lymphopoietin has increasingly gained attention in recent years. Originally, it was implicated in atopic dermatitis and in a variety of other allergic reactions via TH_2_-cells [39,68], but emerging evidence indicates that TSLP is also involved in chronic inflammatory and autoimmune disorders (e.g., psoriasis) and in several cancers [69]. Overexpression of the major epidermal proinflammatory cytokine IL-1α is positively correlated with symptom exacerbation and disease progression in psoriasis, atopic dermatitis, neutrophilic dermatoses, skin phototoxicity and skin cancer [70].

The functionality of the herein presented model is demonstrated by the robust increase of typical inflammatory cytokines that could be modulated by the topical application of DXM. Mass spectrometric analyses of tissue extracts revealed that higher amounts of DXM reached viable layers in SP-treated skin than in untreated skin. This was the case for both the cream and the nanocarrier-based formulation.

Despite the superiority of CMS-NCs, both tested formulations suppressed cytokine production equally. Anti-inflammatory effects of the vehicles cannot be ruled out, especially as cream formulations can per se exert barrier-stabilizing properties when applied over time [71]. The herein observed pronounced short-term effects after application of the ultra-potent corticosteroid DXM formulated in cream or nanocarriers in an aqueous solution provide the basis for wider screening experiments on smaller tissue samples. Rather than focusing on tissue collection for the herein presented protein, RNA and drug extracts, as well as on immunostaining, incorporation of ex vivo dermal microdialysis into the recently presented approach [72] could enable an efficient comparison of different compounds, concentrations or formulations in parallel.

In conclusion, we suggest that the herein presented approach could improve the selection of promising drug delivery strategies at early stages of development. The addition of enzymes is inexpensive when compared to the cytokine cocktails that are frequently used in inflammation research, enables biomarker analyses that are not confounded by external cytokine additions and addresses practically relevant alterations found in diseased skin barriers, which affect penetration rates.

## Supplementary Materials

The following are available online at https://www.mdpi.com/1999-4923/12/9/862/s1, Figure S1: Histological assessment of ex vivo human skin treated with SP. Figure S2: Influence of SP treatment on epidermis cell proliferation in ex vivo human skin. Figure S3: Expression of pro-inflammatory markers IL 6, IL 8, TSLP and IL 1α in ex vivo human skin. Figure S4: Relative expression profiles of pro-inflammatory mediators in ex vivo human skin after SP treatment.
