# Supplementary material for: Serine Protease-Mediated Cutaneous Inflammation: Characterization of an Ex Vivo Skin Model for the Assessment of Dexamethasone-Loaded Core Multishell-Nanocarriers
Source: Pharmaceutics. 2020 Sep 10;12(9):862. doi: 10.3390/pharmaceutics12090862 (PMC7558872; doi:10.3390/pharmaceutics12090862)
Supplement: Supplementary file 1 [file pharmaceutics-12-00862-s001.pdf]

pharmaceutics

Serine protease-mediated cutaneous inflammation: Characterization of an *ex vivo* skin model for the assessment of dexamethasone-loaded core multishell-nanocarriers

Frombach J.[1], Rancan F.[1], Kübrich K.[1], Schumacher F.[2], Unbehauen M.[3], Blume-Peytavi U.[1], Haag R.[3], Kleuser B.[2], Sabat R.[4], Wolk K.[4] and Vogt A.[1]

[1] Clinical Research Center for Hair and Skin Science, Department of Dermatology, Venereology and Allergy, Charité-Universitatsmedizin Berlin, corporate member of Freie Universitaet Berlin, Humboldt-Universitaet zu Berlin, and Berlin Institute of Health, Germany.
[2] Institute of Nutritional Science, University of Potsdam, Potsdam, Germany
[3] Organic Chemistry, Institute of Chemistry and Biochemistry, Freie Universitaet Berlin, Berlin, Germany
[4] Psoriasis Research and Treatment Center, Department of Dermatology, Venerology and Allergy/Institute for Medical Immunology, Charité-Universitaetsmedizin Berlin, corporate member of Freie Universitaet Berlin, Humboldt-Universitaet zu Berlin, and Berlin Institute of Health, Germany.

Corresponding Author: annika.vogt@charite.de

Supplemental material

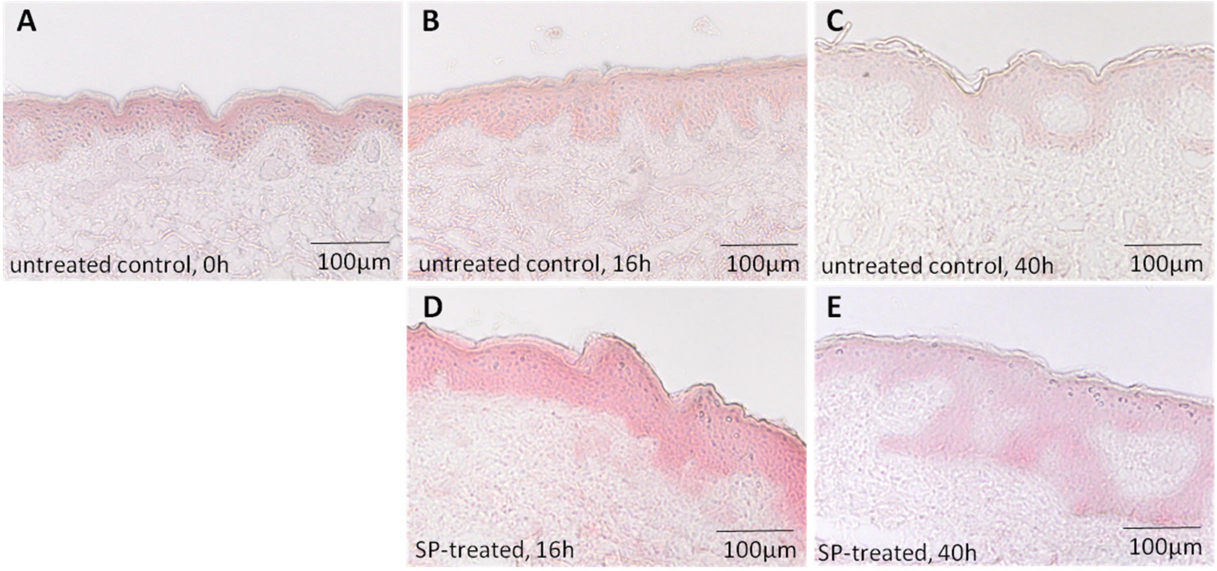

Figure S1: Histological assessment of *ex vivo* human skin treated with SP. (A-E) Images of representative HE-stained untreated (A-C) and SP-treated (D,E) skin sections show a homogeneous histopathological structure of all skin samples over incubation period of 16 and 40 h.

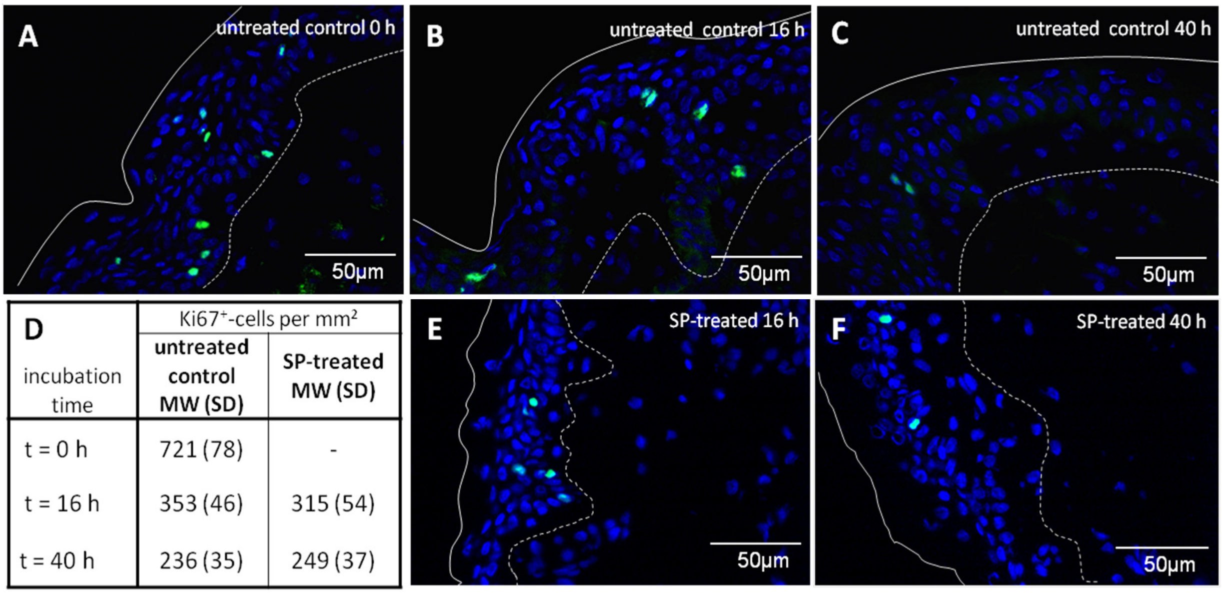

D

incubation time	Ki67⁺-cells per mm² untreated control MW (SD)	Ki67⁺-cells per mm² SP-treated MW (SD)
t = 0 h	721 (78)	-
t = 16 h	353 (46)	315 (54)
t = 40 h	236 (35)	249 (37)

Figure S2: Influence of SP treatment on epidermis cell proliferation in *ex vivo* human skin. Immunofluorescence images of untreated (A-C) and SP-treated skin (E, F) sections were prepared after 0 h, 16 h and 40 h of incubation. Ki67⁺-cells are stained green and cell nuclei blue. **(D)** The number of proliferating cells counted per mm² skin (n=3).

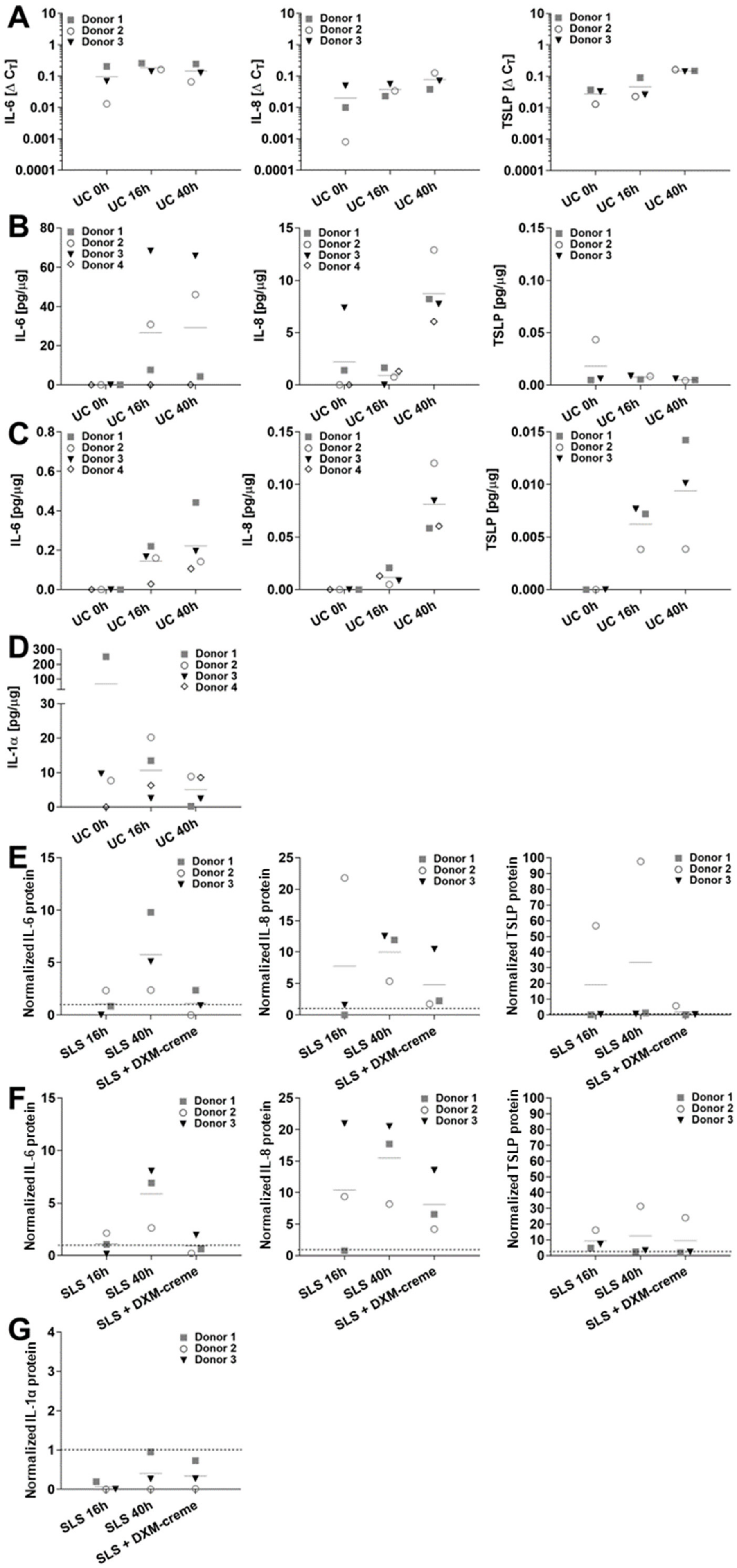
A
B
C
D
E
F
G
Donor 1
Donor 2
Donor 3
Donor 4
IL-6 [Δ C_T]
IL-8 [Δ C_T]
TSLP [Δ C_T]
IL-6 [pg/μg]
IL-8 [pg/μg]
TSLP [pg/μg]
IL-1α [pg/μg]
Normalized IL-6 protein
Normalized IL-8 protein
Normalized TSLP protein
Normalized IL-1α protein
UC 0h
UC 16h
UC 40h
SLS 16h
SLS 40h
SLS + DXM-creme

Figure S3: Expression of pro-inflammatory markers IL-6, IL-8, TSLP and IL-1α in *ex vivo* human skin. (**A**) mRNA-quantification of untreated control (UC) samples by individual qPCR assays: Target gene expressions were normalized to housekeeping gene (hypoxanthine-guanine phosphoribosyltransferase, HPRT) expression. (**B-D**) Quantification of protein of untreated control samples (UC) in different skin layers (B = dermis, C = epidermis, D = superficial *stratum corneum*) by individual ELISA assays: results were normalized to the total protein content of each lysate. (**E-G**) Quantification of protein expression of sodium laryl sulfate (SLS)-treated samples in different skin layers. Short lines indicate the means of the different donors for each sample (E = dermis, F = epidermis, G = superficial *stratum corneum)* by individual ELISA assays: results were normalized to the total protein content of each lysate and depicted as multiples of untreated controls for the same incubation period. Spotted lines indicate the expression level of untreated controls (at y = 1).

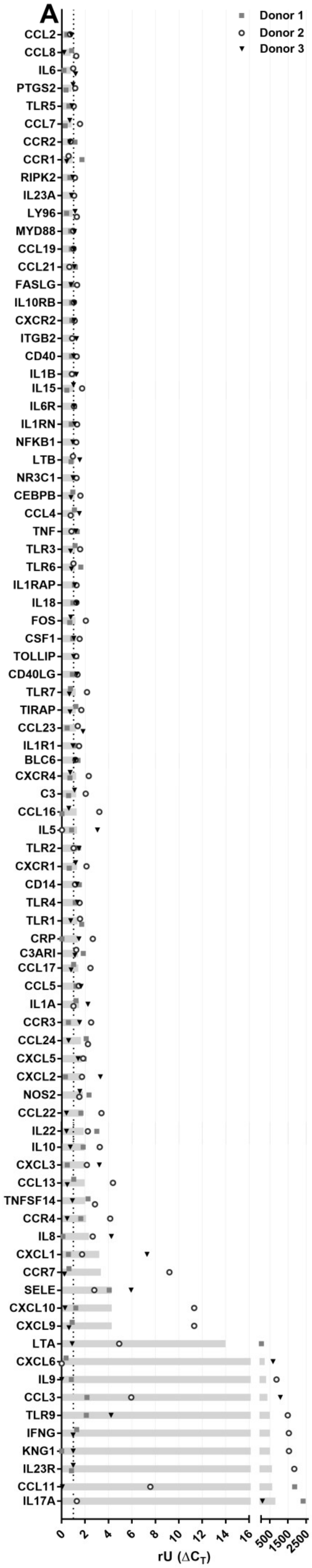

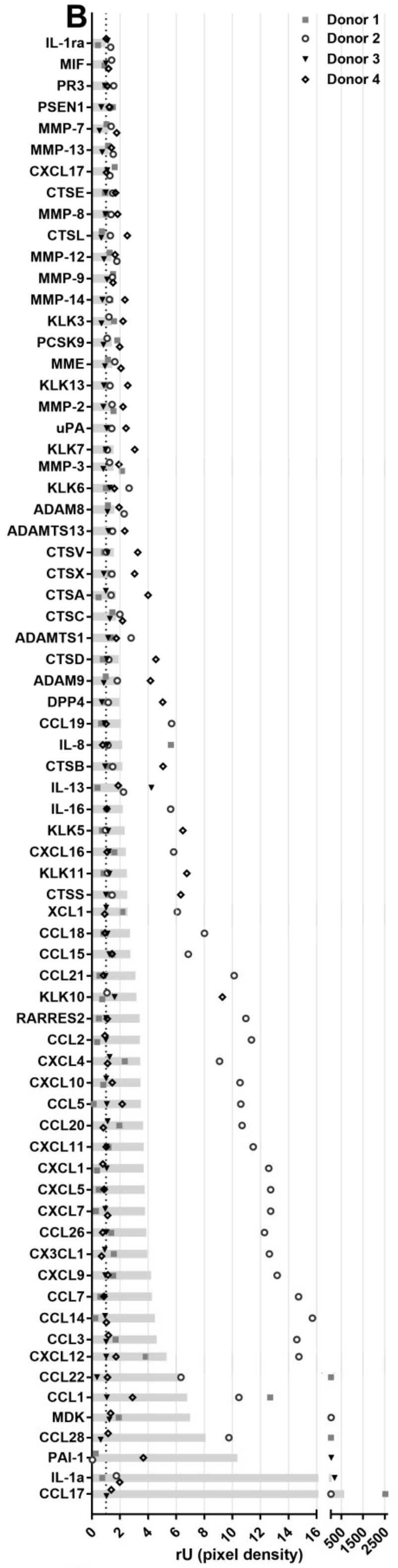

C

each individual rel. mRNA-expression (donor 1-3) > 1	each individual rel. protein-expression (donor 1-4) > 1
BLC6	ADAMTS13
C3ARI	ADAMTS1
CCL3	CCL1
CCL5	CCL17
CD14	CCL3
CXCL5	CTSC
IL17A	CXCL11
IL1RAP	CXCL12
IL6R	CXCL16
NOS2	CXCL17
SELE	CXCL4
TLR4	IL-16
TLR9	MDK
TOLLIP	uPA

Figure S4: Relative expression profiles of pro-inflammatory mediators in *ex vivo* human skin after SP treatment. All data of each donor were depicted as multiples of untreated controls for the same incubation period. Bars represent the means of the different donors for each sample. Spotted lines indicate the expression level of untreated controls (at y = 1). (A) Quantification of mRNA (84 species) by Inflammatory Response & Autoimmunity RT^2 Profiler PCR Array after 16 h incubation, normalized to the expression of the housekeeping gene hypoxanthine-guanine phosphoribosyltransferase (HPRT). (B) Quantification of protein (70 out of 101 analytes) in the epidermal layer by cytokine-, chemokine- and protease-Proteome Profiler after 40 h incubation: Profiler membranes were loaded with 100 µg total protein of epidermis lysates. (C) Table of upregulated inflammatory mediators which were found by mRNA/proteome profiling (shown in (A/B) and meet definition: individual values of all tested donors >1.

© 2020 by the authors. Submitted for possible open access publication under the terms and conditions of the Creative Commons Attribution (CC BY) license (http://creativecommons.org/licenses/by/4.0/).
